# *POLR3A*-related syndrome complicated with cerebral abscesses: a case report and literature review

**DOI:** 10.3389/fgene.2026.1668022

**Published:** 2026-02-05

**Authors:** Mengyao Zhou, Lingli Hou, Huijuan Fan, Yuanfang Duan, Xinye Xie, Haohao Wu, Hanmin Wang, Wei Zhang, Kang Du

**Affiliations:** 1 Department of Endocrinology and Metabolism, Yunnan Qujing Central Hospital (Qujing First People’s Hospital), Qujing, Yunnan, China; 2 Department of Ultrasound, Yunnan Qujing Central Hospital (Qujing First People’s Hospital), Qujing, Yunnan, China; 3 Department of Neurology, Yunnan Qujing Central Hospital (Qujing First People’s Hospital), Qujing, Yunnan, China

**Keywords:** abscesses, brain atrophy, homozygous variant, leukodystrophy, POLR3A gene

## Abstract

**Background:**

*POLR3A* gene-related syndrome is a complex genetic disorder with diverse clinical manifestations. Understanding its characteristics is crucial for diagnosis and management. Previous studies have reported various aspects of this syndrome, yet a comprehensive analysis of different Variant sites and their associated phenotypes remains necessary.

**Case report:**

This study presents a case of *POLR3A*-related syndrome in a pediatric patient. Symptom onset occurred after 2 years of age, initially presenting with gait disturbance. As the disease progressed, gait instability worsened progressively and was accompanied by dysarthria, intellectual developmental impairment, and tremor. Subsequent neuroimaging revealed multiple intracerebral infectious lesions with abscess formation. Whole-genome sequencing identified a homozygous c.1771-6C>G variant in the *POLR3A* gene. This variant has been previously reported as pathogenic at this locus; however, the complication of multiple intracerebral infections and abscess formation represents a previously unreported manifestation. It is noteworthy that the parents of the proband were consanguineous (first-degree relatives).

**Conclusion:**

A review of 60 unrelated probands with *POLR3A*-related syndrome was conducted based on previously published cases. The analysis revealed no significant sex difference in disease occurrence. The median age of onset was approximately 8 years, with common initial symptoms including gait disturbance and cognitive developmental impairment. Neuroimaging findings indicated cerebral atrophy in 31 cases (66.0%) and white matter hypomyelination in 17 cases (34.7%). Among the reported genetic variants, c.1909 + 22G>A was the most prevalent, identified in 19 families (17.8%), followed by c.1771-6C>G in 9 families (6.4%). Furthermore, patients with different variant sites displayed heterogeneity in initial symptoms, clinical presentations, and imaging characteristics. This comprehensive review enhances the understanding of the phenotypic and genotypic spectrum of *POLR3A*-related syndrome.

## Introduction

The *POLR3* gene encodes the subunits of RNA polymerase III (Pol III), including *POLR3A* and *POLR3B*. Pol III is an enzyme complex that plays a role in protein synthesis within cells, particularly by transcribing tRNAs and a few other non-coding RNAs, such as 5S ribosomal RNA (Dieci et al., 2007; Bernard et al., 2011). The *POLR3A* gene is located in the 10q22.3 region, consisting of 6610 base pairs. Variants in this gene include missense Variants, nonsense Variants, splice site Variants, insertions, deletions, and large segment insertions/deletions (Ji et al., 2018). Pathogenic variants of this gene can lead to a spectrum of diseases related to the *POLR3A* gene. Atrouni et al. first documented *POLR3A*-related phenotypic features (Atrouni et al., 2003), while Timmons et al. later reported four cases with the classic triad of hypomyelination, hypogonadotropic hypogonadism, and hypodontia and formally proposed the term “4H syndrome”which is a specific subset of *POLR3A*-related phenotypes (Timmons et al., 2025). The spectrum of diseases in 4H syndrome includes Hypomyelinating Leukodystrophy-7 (HDL7) and Wiedemann-Rautenstrauch syndrome (WRS), which may include neurological manifestations like abnormal tooth development and/or hypogonadism. It is a neurodegenerative disease caused by an autosomal recessive inheritance. The main symptoms include neurologic dysfunction, such as progressive cerebellar dysfunction, muscle tone abnormalities, spasms, and cognitive impairments. Other symptoms may include abnormal tooth development, hypogonadism, endocrine abnormalities, and myopia (Minnerop et al., 2017; Rydning et al., 2019). Rare cases of this disease have been reported in China (Liang et al., 2017; He et al., 2021; Song et al., 2021a; Li et al., 2022). This study reports a case of a patient with *POLR3A* gene-related syndrome and reviews literature to enhance understanding of the disease.

## Case report

A 30-year-old male patient was admitted on 24 June 2023, with a 15-year history of head tremor, an 11-year history of progressively abnormal gait, and a 3-year history of involuntary generalized body tremors. The patient’s symptoms began at age 11 with a dragging gait; however, no abnormalities were noted during uphill walking, squatting, or routine ambulation, though he experienced frequent falls. By age 14, he had developed involuntary head tremors and noticeable anterior-posterior sway during walking. His gait impairment gradually worsened without specific intervention. Over the past 4 years, the patient exhibited involuntary tremors in both upper limbs, particularly during object manipulation, accompanied by limited fine motor control. Previous treatment with muscle tone-modulating medications at another facility yielded no significant improvement. One week prior to admission, he reported intermittent headaches accompanied by fever, with a maximum recorded temperature of 40 °C. There was no loss of consciousness, limb convulsions, or specific treatment administered before presentation. He was admitted through the emergency department under the diagnosis of “central nervous system infection.” Notably, the patient achieved ambulation after the age of 2 but continued to experience occasional falls. His speech was characterized by clumsiness, dysarthria, and rapidity, resulting in reduced intelligibility. Around age 7, family members observed that his intellectual development lagged behind that of his peers, with corresponding academic difficulties. The patient’s parents are consanguineous, and no similar symptoms have been reported among first-degree relatives. The patient has no children. A history of gas poisoning 4–5 months prior was reported, with no significant sequelae.

On admission, the patient’s neurological examination revealed the following: vital signs were within normal limits; height was 152 cm and weight 41 kg. He was alert and fully oriented to person, but disoriented to time and place. His speech was severely dysarthric. While he was only partially cooperative with the examination, he was able to follow simple commands. Cognitive assessment showed impaired calculation ability and reduced comprehension. Cranial nerve examination demonstrated normal visual acuity, pupils equal and round (3.0 mm in diameter) with intact light reflex, full extraocular movements without nystagmus, and bilateral symmetry of the nasolabial folds. Dentition was normal. Motor examination revealed full (5/5) muscle strength and symmetric, normal muscle tone throughout. Deep tendon reflexes, including the patellar reflex, were brisk. Sensory examination was notable for intact pain perception bilaterally; however, further sensory testing was limited due to poor cooperation. Coordination testing showed an unsteady finger–nose test and heel–knee–shin test. No pathological reflexes or signs of meningeal irritation were observed.

**FIGURE 1:**
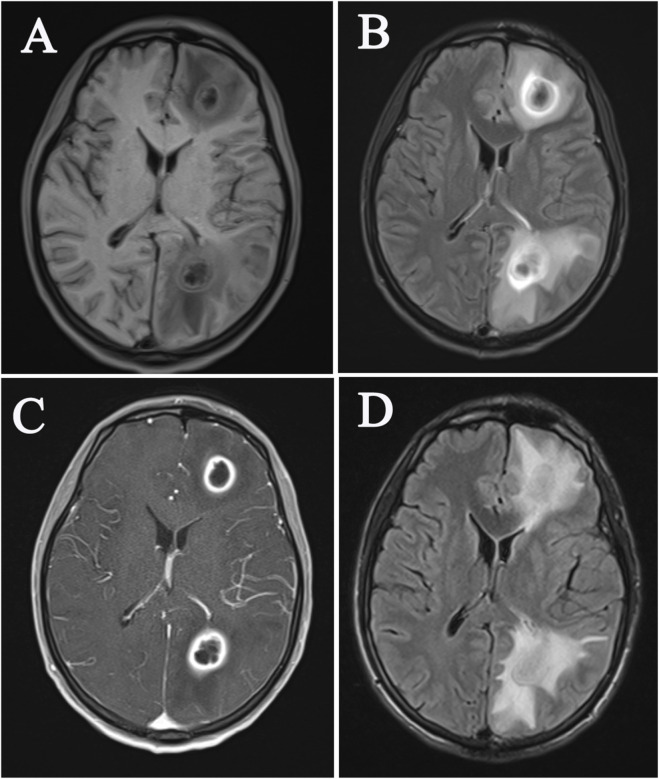
Brain MRI imaging features of patients at initial diagnosis **(A-C)** and after a follow-up examination **(D)**.

Auxiliary examination results: white blood cell count was 12.7 × 10^9/L, neutrophil percentage was 82.2%, and C-reactive protein was 45.3 mg/L. The blood culture showed the detection of Gram-positive bacteria and anaerobic bacteria. Blood routine, urine and stool routine, biochemical indicators, homocysteine, ceruloplasmin, hepatitis, syphilis, HIV, coagulation function, autoimmune antibody spectrum, etc., Were all normal. No abnormalities were found in cardiac ultrasound. Upon admission on 26 June 2023, brain magnetic resonance imaging (MRI) and an enhanced examination indicated multiple infections and abscess formation in the brain (Figures 1A–C). There was no white matter high signal, low myelinization, brain atrophy, or ventricular enlargement seen. “Hereditary ataxia” was considered in this patient, so genetic testing was performed. Whole genome sequencing results showed a homozygous Variant in the *POLR3A* gene, c.1771-6C>G, which has been reported as a pathogenic Variant site before (Hiraide et al., 2020). The patient was ultimately diagnosed with *POLR3A*-related syndrome and cerebral abscess. The patient was treated with piperacillin-tazobactam sodium 4.5 mg Q6h ivgtt for anti-infective therapy and upon discharge, the patient’s headache had improved compared to before. A follow-up brain MRI on 10 July 2023, showed a decrease in the size of the abscess (Figure 1D). Over half a year after discharge, the patient’s headache has eased, but symptoms such as gait instability, dysarthria, tremors, and cognitive impairment still persist.

## Literature review and statistical analysis

This study searched the databases “Pubmed,” “Wanfang Medical,” and “CNKI” using the keywords “*POLR3A*” and “leukoencephalopathy.” A literature review was conducted on previously reported cases of leukoencephalopathy associated with the *POLR3A* gene in both Chinese and English. A total of 60 unrelated pedigrees were reviewed, and a summary of the clinical and genotype information of the index cases in all pedigrees was compiled. All statistical analyses were performed using GraphPad Prism software (version 9.0). Descriptive statistical methods were employed to systematically summarize and compare the clinical phenotypes and MRI characteristics between patients carrying the c.1909 + 22G>A variant and those with non-c.1909 + 22G>A mutations, with the aim of identifying potential phenotypic variations and disease patterns. Categorical variables were expressed as frequencies and percentages. Based on data distribution and sample size, group comparisons for categorical variables were conducted using the chi-square test, continuity correction test, or Fisher’s exact test, as appropriate. All statistical tests were two-tailed, and a *p*-value <0.05 was considered statistically significant.

After reviewing literature (Table 1), it was determined that among 60 probands from different families, the male-to-female gender ratio was 26:29, with a median age of 8.0 years (ranging from 0 to 30 years old). Gait disturbances were the initial symptoms in 26 cases (55.3%), while developmental abnormalities were presenting symptoms in 10 cases (21.3%), including 2 cases (4.2%) with gonadal developmental abnormalities and 1 case (2.1%) with facial dysmorphism. Clinical manifestations included tremors in 34 cases (56.7%), eye tremors in 24 cases (40.0%), head and body tremors in 23 cases (38.3%), ataxia in 31 cases (51.7%), gait abnormalities in 31 cases (51.7%), dental abnormalities in 30 cases (50.0%), limb spasticity in 23 cases (38.3%), speech disorders in 21 cases (35.0%), cognitive impairment in 19 cases (31.7%), muscle tone disorders in 14 cases (23.3%), epilepsy in 6 cases (10.0%), and gonadal developmental abnormalities in 5 cases (8.3%).

**TABLE 1:** Literature review of probands with *POLR3A* gene-related syndrome probands previously reported.

Family serial number	Age at onset (y.)	Disease course (y.)	Gender	Symptom at onset	Clinical preservation and symptom	Brain MRI	Variant 1	Variant 2	Author, year
1	12	43	M	gait clumsiness, gait clumsiness and recurrent falls	gait ATX, limb ATX, SA (gait), CD, head titubation, NYST, DENT	mild cerebellar atrophy	c.1909 + 22G>A	c.3337-11T>C	Fellner et al. (2021)
2	16	2	F	gait instability, coughing when drinking water	hypogonadism, CI, gait ATX	myelin sheath dysplasia in bilateral cerebral hemisphere, brain atrophy, thin corpus callosum, small pituitary, uneven enhancement, ventricular enlargement	c.3013C>T	c.1757C>T	Yang et al. (2023)
3	18	22	M	gait clumsiness	gait ATX, SA (gait), lower limb ATX	significant abnormal signals in bilateral superior cerebellar peduncle and midbrain	c.1909 + 22G>A	c.1787C>T	Infante et al. (2020)
4	30	18	M	hand tremor	gait ATX, SA (gait), limb ATX, mild CD, extensor plantar	significant abnormal signals in bilateral superior cerebellar peduncle and midbrain	c.1909 + 22G>A	c.592G>T	
5	30	30	F	gait clumsiness	gait ATX, SA (gait), lower limb ATX	significant abnormal signals in bilateral superior cerebellar peduncle and midbrain	c.1909 + 22G>A	c.1993dupT	
6	13	45	F	gait linstability	gait ATX, SA (gait), limb ATX, mild CD, extensor plantar, DENT	significant abnormal signals in bilateral superior cerebellar peduncle and midbrain	c.1909 + 22G>A	c.646-687_1185 + 844del	
7	18	35	M	gait clumsiness	gait ATX, SA (gait), limb ATX	significant abnormal signals in bilateral superior cerebellar peduncle and midbrain	c.1909 + 22G>A	c.646-687_1185 + 844del	
8	27	5	F	gait clumsiness	gait ATX, SA (gait), lower limb ATX, extensor plantar	significant abnormal signals in bilateral superior cerebellar peduncle and midbrain	c.1909 + 22G>A	c.646-687_1185 + 844del	
9	0.8	1.7	F	reduced motor ability, nystagmus, motor ataxia, dysarthria, spastic tetraplegia	ATX, NYST, CI, CD, SPAST-TET, DENT, polytrichia, hypermyotonia, DYSPHAG	cerebella, corpus callosum atrophy, white matter hypomyelination	c.1771-6C>G	c.2611del	Wu et al. (2019)
10	0.6	0.9	M	developmental delay	DD, SPAST-PARA, MYODYS, progeroid facial appearance	NA	c.2005C>T	c.1771-7C>G	Majethia and Girisha, (2021)
11	0.5	10.5	F	developmental delay	DD, mobility limitation, ATX, aphasia, severe mental retardation, visual dysplasia, stereotypic hand movements, active tendon reflex, hypermyotonia, mild SA	cerebella, corpus callosum atrophy, white matter hypomyelination	c.346A>G	c.1745G>A	Khalifa and Naffaa, (2015)
12	30	36	F	progressive gait disorder	gait ATX, SPAST-PARA (gait), hypermyotonia (Babinski sign positive)	cerebellum and cervical spinal cord atrophy	c.1909 + 22G>A	c.3839dupT	Ruggiero et al. (2020)
13	2	NA	M	NA	DD, NYST, CI, TR, upper motor neuron signs, cerebellar signs, DENT	NA	c.1674C>G	c.3742insACC	Bernard et al. (2011)
14	1	NA	M	NA	DD, seizure, optic atrophy, NYST, DYSPHAG, TR, upper motor neuron signs, cerebellar signs, hypersalivation	NA	c.2015G>A		
15	3	NA	M	NA	CI, optic atrophy, NYST, DYSPHAG, upper motor neuron signs, cerebellar signs, hypersalivation	NA	c.2015G>A		
16	5	NA	F	NA	CI, optic atrophy, upper motor neuron signs, cerebellar signs, hypersalivation	NA	c.2015G>A		
17	13	NA	F	NA	CI, seizure, DYSPHAG, upper motor neuron signs, cerebellar signs, hypersalivation	cerebellar atrophy, white matter hypomyelination	c.2554A>G	c.2711-1G>A	
18	0	NA	F	NA	DD, CI, DYSPHAG, upper motor neuron signs, cerebellar signs, hypersalivation	cerebellar atrophy, white matter hypomyelination	c.2324A>T	c.1114G>A	
19	13	NA	M	NA	CI, DENT, vertical gaze limitation, DYSPHAG, upper motor neuron signs, cerebellar signs	cerebellar atrophy, white matter hypomyelination	c.2830G>T	c.3013C>T	
20	12	NA	F	NA	CI, DENT, vertical gaze limitation, DYSPHAG, upper motor neuron signs, cerebellar signs	cerebellar atrophy, white matter hypomyelination	c.2554A>G	c.2711-1G>A	
21	1	NA	F	NA	CI, DD, DENT, upper motor neuron signs, cerebellar signs, TR	cerebellar atrophy, white matter hypomyelination	c.4006C>T	c.1907C>A	
22	12	NA	F	NA	CI, upper motor neuron signs, cerebellar signs, TR	NA	c.2003 + 18G>A		
23	3	NA	F	NA	CI, DD, DENT, upper motor neuron signs, cerebellar signs, TR	NA	c.418C>T	c.2554A>G	
24	2	NA	M	NA	CI, DD, DENT, vertical gaze limitation, upper motor neuron signs, cerebellar signs, TR, hypersalivation	NA	c.2171G>A		
25	5	1	F	developmental delay	local lipoatrophy, alopecia areata, osteopenia, progeroid facial appearance, DENT	NA	c.3568C>T	c.3337-11T>C	Temel et al. (2020)
26	1	28	F	esotropia and action tremor	ATX, CD, DENT, dysaudia, NYST, intention TR, postural seizures	white matter hypomyelination, brainstem, cerebellum and corpus callosum atrophy	c.930G>C	c.2411T>C	Shimojima et al. (2014)
27	19	15	F	gait disorder, amenorrhea, progressive cognitive impairment	ATX, CD, NYST, limb SA, DYSK	the corpus callosum, cortex/subcortex, brain stem and cerebellum atrophy, supratentorial ventricular system dilatation, white matter hypomyelination	c.2325C>G	c.2554A>G	Campopiano et al. (2020)
28	15	26	F	dental developmental abnormalities, amenorrhea	amenorrhea, movement disorder, DENT, gait ATX, cerebellar ATX, limb SA, tonic seizure, DYSPHAG, osteoporosis	white matter hypomyelination, brainstem, cerebellum and corpus callosum atrophy	c.2554A>G	c.2668G>T	Furukawa et al. (2021)
29	8	0	M	cerebellar dysarthria	CD, ATX, hypomyotonia, TR	bilateral symmetric atrophy, increased sig-nal of the caudate nucleus and the putamen	C.1771-6C>G	c.791C>T	Hiraide et al. (2020)
30	1.5	NA	F	dysstasia	gait ATX, CD, ophthalmoparesis, MYODYS, DENT, mandibular underdevelopment, hypomyotonia	diffuse brain atrophy	c.1771-6C>G	c.2671C>T	
31	18	35	F	gait ataxia	gait ATX, ATX, CD, DD, DENT	white matter hypomyelination, corpus callosum, cerebellum atrophy, thoracic spinal cord thinning	c.928T>A	c.3295C>T	Li et al. (2022)
32	1	2	F	developmental delay	DD, coughing when drinking, mobility limitation, MYODYS	abnormal signals in lentiform nucleus, putamen and caudate nucleus	c.1980 G>C	c.1771-6 C>G	He et al. (2021)
33	1.3	2.7	F	developmental delay	DD, seizure, MYODYS, NYST	abnormal signals in caudate nucleus, lentiform nucleus and bilateral paraventricular	c.2044C>T	c.1771-7C>G	
34	0.5	1.5	M	gait linstability	DD, gait ATX, DENT, CD, NYST, TR	diffuse abnormal signals in bilateral large and small cerebral hemisphere white matter area	c.3858C>A	c.3226G>A	Song et al. (2021b)
35	5.7	0.3	M	gait linstability, delayed development of language	DD, gait ATX, DENT, optic atrophy, NYST, intention TR	widely symmetrical white matter lesions on both sides of the cerebral hemisphere	c.1781T>G	c.2693delT	Liang et al. (2017)
36	0.3	36.7	F	facial deformity	facial deformity, DD, hearing abnormality, DENT, severely cachexic appearanc	abnormal signal in cerebellar	c.3336G>A		Lessel et al. (2022)
37	1.5	15.5	M	delayed psychomotor development and absence of language, gait ataxia	ATX, CD, DENT, DYSPHAG, NYST, gait ATX, ptosis	corpus callosum, cerebellum atrophy, white matter hypomyelination	c.1795C>A	c.328A>G	Musumeci et al. (2022)
38	1.5	4.5	M	gait ataxia	dysontogenetic, seizure, MYODYS, DYSPHAG, CD	abnormal signal in striatum	c.1771-6C>G	c.4037G>A	Nikkhah and Rezakhani, (2022)
39	19	56	M	gait ataxia	DYSK, gait ATX, NYST, CD, pyramidal signs, cerebellar signs, head TR, sensory peripheral neuropathy	NA	c.1909 + 22G>A		Sytsma et al. (2022)
40	15	27	F	developmental delay	ATX, DENT, amenorrhea	diffuse cortical atrophy, white matter hypomyelination	c.1911 + 18C>T		Yang et al. (2019)
41	0.5	1	F	nystagmus	ATX, DD, DENT, NYST, hearing abnormality, mandibular underdevelopment	white matter hypomyelination, diffuse atrophy	c.2423G>A		Tewari et al. (2018)
42	26	8	F	gait ataxia	ATX, CD, mobility limitation, DENT, NYST, static TR	abnormal signals around the ventricle, frontal lobe and temporal lobe	c.4044C>G	c.1186-2A>G	Sun et al. (2023)
43	4	29	M	dyskinesia	hypogonadism, CI, DENT, cerebellar ATX, intelligence decline	cerebella, corpus callosum atrophy, white matter hypomyelination, cerebellar abnormal signal	c.2554A>G	c.3745A>C	Terao et al. (2012)
44	8	48	M	cerebellar dysarthria, gait ataxia	ATX, movement disorder, NYST, bilateral symmetric rigidity (right hand/lower limb), right wrist gear phenomenon	a mild small brain, with bilateral symmetric atrophy of the caudate nucleus and putamen and associated increased signal, focal symmetrical signal changes in the medial red nucleus area and the third brain nerve axis, the white matter was of normal volume and signal	c.1771-6C>G		Azmanov et al. (2016)
45	7	24	M	speech disturbances	MYODYS, gait instability, DYSPHAG, dysmelia, intelligence decline, extensor plantar	bilateral symmetric atrophy and increased signal of the caudate nucleus and putamen, with prominence of the lateral ventricular frontal horns as a consequence, and focal bilateral symmetric signal change in the region of the medial red nucleus intra-axial course of the third cranial nerve	c.1771-6C>G		
46	NA	NA	NA	gait ataxia, cerebellar dysarthria, tremor	cerebellar TR, DENT	a selective involvement of the corticospinal tracts, which was particularly evident at the level of the posterior limbs ofthe internal capsule as T2-hyperintense signal	c.1048 + 1G>A	c.128913A>C	La et al. (2016)
47	NA	NA	NA	spasticity and diplegic gait	cerebellar TR, pyramidal signs, SA, severe dystonic TR, DENT	a selective involvement of the corticospinal tracts, which was particularly evident at the level of the posterior limbs of the internal capsule as T2-hyperintense signal, focal, partially confluent, T2-hyperintense white matter abnormalities located in the deep frontal and parietal white matter, suggesting partial hypomyelination	c.2710 G>A		​
48	NA	NA	NA	spasticity and diplegic gait	cerebellar TR, pyramidal signs, SA, severe dystonic TR, DENT	moderate to severe cerebellar atrophy was variably associated with nonspecific T2-hyperintense white matter abnormalities or thinning of the corpus callosum. Focal, partially confluent, T2-hyperintense white matter abnormalities located in the deep frontal and parietal white matter, suggesting partial hypomyelination	c.1771-6C>G	c.3205C>T	
49	NA	NA	NA	NA	pyramidal signs, SA	moderate to severe cerebellar atrophy was variably associated with nonspecific T2-hyperintense white matter abnormalities or thinning of the corpus callosum.focal, partially confluent, T2-hyperintense white matter abnormalities located in the deep frontal and parietal white matter, suggesting partial hypomyelination	c.2381A>C	c.-35C>G	
50	NA	NA	NA	gait ataxia, cerebellar dysarthria, tremor	pyramidal signs, SA, DENT	a selective involvement of the corticospinal tracts, which was particularly evident at the level of the posterior limbs of the internal capsule as T2-hyperintense signal	c.1909 + 22G>A	c.2549A>G	
51	14	51	F	gait linstability	TRE-ATX, limbs tendon reflex weakened, lower limb weakness/atrophy, CD, limb ATX, foot deformity, postural TR, head/neck titubation, hypoesthesia, urinary urgency	SCP high signal, cervical spinal cord thinning, cerebellar hemisphere and vermis atrophy	c.1909 + 22G>A	c.3655G>T	Rydning et al. (2019)
52	12	35	M	gait linstability	TRE-ATX, lower limb SA, limb tendon reflex reduction, lower limb weakness/atrophy, NYST, CD, limb ATX, CI, myopia, postural TR, head/neck titubation, hypoesthesia, urinary urgency, foot deformity	SCP high signal, cervical spinal cord thinning, cerebellar vermis atrophy	c.1909 + 22G>A	c.3655G>T	
53	17	27	M	stiff legs	TRE-ATX, lower limb SA, limb tendon reflex reduction, lower limb weakness/atrophy, MYODYS, NYST, CD, limb ATX, postural TR, head/neck titubation	SCP high signal, cervical spinal cord thinning, cerebellar hemisphere and vermis atrophy	c.1909 + 22 G>A	c.3655G>T	​
54	13	33	M	gait linstability	cHSP, lower limb SA, limb tendon reflex reduction, lower limb weakness/atrophy, MYODYS, postural TR, limb ATX, head/neck titubation, hypoesthesia, CI, foot deformity	SCP high signal, cervical spinal cord thinning, cerebellar vermis atrophy	c.1909 + 22 G>A	c.3655G>T	
55	30	15	M	gait linstability	TRE-ATX, lower limb SA, limb tendon reflex reduction, lower limb weakness/atrophy, NYST, upper limb ATX, DENT, hypoesthesia, urinary urgency, foot deformity	SCP high signal, cervical spinal cord thinning, cerebellar vermis atrophy	c.1909 + 22 G>A	c.3655G>T	
56	10	55	F	clumsy	TRE-ATX, lower limb SA, limb tendon reflex reduction, lower limb weakness/atrophy, NYST, CD, limb ATX, MYODYS, head/neck titubation, DENT, myopia, postural TR, hypoesthesia	NA	c.1909 + 22 G>A	c.3655G>T	
57	11	46	M	stiff legs	cHSP, lower limb SA, limb tendon reflex reduction, lower limb weakness/atrophy, NYST, lower limb ATX, DENT, myopia, hypoesthesia, urinary urgency, scoliosis	SCP high signal, cervical spinal cord thinning, cerebellar hemisphere and vermis atrophy	c.1909 + 22 G>A	c.1682G>A	
58	17	28	M	gait linstability	TRE-ATX, lower limb SA, limb tendon reflex reduction, lower limb weakness/atrophy, NYST, CD, limb ATX, DENT, postural TR, MYODYS, myopia, hypoesthesia, urinary urgency, scoliosis, hypogonadism	SCP high signal, cervical spinal cord thinning, cerebellar vermis atrophy	c.1909 + 22 G>A	c.1378_ 1380del	
59	5	24	F	gait linstability	TRE-ATX, limb tendon reflex reduction, NYST, limb ATX, DENT, MYODYS, hypoesthesia, foot deformity, head/neck titubation, CI	SCP high signal, cervical spinal cord thinning, cerebellar vermis atrophy	c.1909 + 22 G>A	c.1378_ 1380del	
60	4	41	M	gait linstability	cHSP, lower limb SA, limb tendon reflex reduction, lower limb weakness/atrophy, CD, NYST, limb ATX, DENT, MYODYS, myopia, hypoesthesia, postural TR, head/neck titubation	SCP high signal, cervical spinal cord thinning, cerebellar vermis atrophy	c.1771-6 C>G		

Abbreviation: ATX, ataxia; SA, spasticity; TR, tremor; CD, cerebellar dysarthria; CI, cognitive impairment; DENT, dental abnormalities; DD, developmental delay; NYST, nystagmus; DYSPHAG, dysphagia; SPAST-TET, spastic tetraplegia; SPAST-PARA, spastic paraplegia; MYODYS, myodystonia; DYSK, dyskinesia; CEREBELL-ATRO, cerebellar atrophy; CORP-CALL-ATRO, corpus callosum atrophy; CORP-CALL, corpus callosum; WM-HYPOMYO, white matter hypomyelination; SCP, superior cerebellar peduncles; TRE-ATX, Tremor-ataxia; cHSP, complex hereditary ataxia and spastic paraparesis; F, female; M, male; NA, not available.

Compound heterozygous Variants were found in a total of 47 cases (78.3%), while homozygous Variants were found in 13 cases (21.7%). The most common Variant was the c.1909 + 22G>A Variant, found in a total of 19 families (17.8%), followed by the c.1771-6 C>G Variant in 9 families (6.4%), and the c.2554A>G Variant in 6 families (5.6%). Other types of Variants were less common. Of the 49 probands, complete head MRI scans were performed in a total of 31 cases (66.0%). Among them, 25 cases (51.0%) showed cerebellar atrophy, 17 cases (34.7%) showed white matter demyelination, 10 cases (20.4%) had abnormal signals in the basal ganglia, and 6 cases (12.2%) showed diffuse atrophy. Among the homozygous Variant patients, 3 cases (42.9%) showed white matter demyelination and 3 cases (42.9%) had abnormal signals in the basal ganglia. Among the patients with compound heterozygous Variants, 15 cases (35.7%) showed white matter demyelination and 24 cases (57.1%) showed cerebellar atrophy.

Among the probands with the Variant site c.1909 + 22G>A, the male-to-female ratio is 11:7, with a median age of onset at 17.0 years old (5–30 years old). Of these cases, 18 cases (94.7%) had compound heterozygous Variants, while 1 case (5.3%) had a homozygous Variant. The most common initial symptom was gait abnormality in 16 cases (84.2%), followed by lower limb stiffness in 2 cases (10.5%) and tremor in 2 cases (10.5%). Clinical manifestations included gait abnormalities in 17 cases (89.5%), ataxia in 16 cases (84.2%), limb spasm in 15 cases (78.9%), dysarthria in 9 cases (47.4%), and dental anomalies in 8 cases (42.1%). Brain MRI revealed significant abnormal signals in the cerebellar foot and midbrain in 14 cases (82.4%), and cerebellar atrophy in 10 cases (58.8%). A comparative analysis of clinical and MRI manifestations between patients carrying the c.1909 + 22G>A variant and those with other genotypes (non-c.1909 + 22G>A) was conducted using Fisher’s exact test, which revealed a distinct phenotypic profile associated with the c.1909 + 22G>A variant. Notably, abnormalities of the superior cerebellar peduncles (SCP) were significantly more common in the variant group (82.4%, 14/17), whereas white matter hypomyelination and bulbar symptoms were conspicuously absent (0/17). Furthermore, key clinical symptoms including sensory impairment and muscle atrophy were both present in 42.1% (8/19) of the variant patients, a prevalence that was significantly higher than that in the non-c.1909 + 22G>A group.

Among the first founders with the Variant site c.1771-6C>G, the male-to-female ratio is 5:3, and the median age of onset is 2.75 years (ranging from 0.8 to 8 years). Of these founders, 6 cases (66.7%) have compound heterozygous Variants and 3 cases (33.3%) have homozygous Variants. Additionally, 4 cases (44.4%) presented with gait abnormalities as initial symptoms, while 4 cases (44.4%) had dysarthria. Clinical manifestations included muscle tone disorders in all cases, tremors in 5 cases (55.6%), dental abnormalities in 4 cases (44.4%), ataxia in 4 cases (44.4%), developmental disorders in 3 cases (60.0%), and developmental delay in 2 cases (22.2%). Brain MRI showed abnormal signals in the striatum in 6 cases (66.6%), diffuse brain atrophy in 3 cases (33.3%), cerebellar atrophy in 2 cases (22.2%), and leukodystrophy in 2 cases (22.2%). The average age of onset for patients with compound heterozygous Variants was 3.0 years, while patients with homozygous Variants had an average age of onset of 6.3 years.

In carriers with the c.2554A>G Variant site, there is a ratio of 1 man to 5 women, with a median age of onset of 12.5 years (ranging from 3 to 19 years). All individuals have compound heterozygous Variants. Of these cases, 2 (33.3%) initially presented with menopause as a symptom. Cognitive impairment was seen in 4 cases (66.7%), abnormal tooth development in 4 cases (66.7%), and ataxia in 3 cases (50.0%). In all four cases, brain MRI scans revealed cerebellar atrophy and white matter demyelination.

## Discussion

Genes such as *POLR3A*, *POLR3B*, *POLR3C*, and *POLR3K* encode RNA polymerase III (RNAPol III), which is responsible for synthesizing small RNAs. Variants in these genes can impact the function of non-coding RNAs that are crucial in cytoplasmic protein synthesis, myelin sheath development, and maintenance in the brain. This can further affect cell growth, differentiation, and apoptosis (Song et al., 2021a). Among these genes, Variants in *POLR3A* are believed to be more common and can lead to more severe clinical manifestations (Wolf et al., 2014). The different genotypes of the *POLR3A* gene are closely linked to the age of onset (Dum et al., 2010) and severity of *POLR3A*-related syndromes, with earlier onset potentially resulting in more severe clinical manifestations (Song et al., 2021a). The affected systems of this disease include the central nervous system and endocrine system and may be related to the high expression of the *POLR3A* gene in the central nervous system, gonads, and reproductive cells (Pelletier et al., 2021).

This study, conducted through literature review, found that there are slightly more female patients than male patients in *POLR3A* gene-related syndromes. The disease typically presents at an early age with gait disorders and developmental abnormalities as initial symptoms. These developmental abnormalities primarily include motor developmental issues, along with occasional gonadal developmental abnormalities, dental developmental abnormalities, and facial dysmorphism. As the disease progresses, additional symptoms such as ataxia, cognitive impairment, epilepsy, tremors, dysarthria, and swallowing difficulties may manifest. Swallowing difficulties and dysarthria are believed to be caused by reduced myelination levels in the medulla oblongata (Bernard et al., 2010), and may also be a result of weak pharyngeal muscle strength or impaired pharyngeal muscle tone. Ataxia in patients may be associated with cerebellar atrophy, white matter lesions, and midbrain involvement (Bernard et al., 2011; Azmanov et al., 2016).

Additionally, common peripheral nervous system manifestations of the disease include dental hypoplasia and hypogonadism. Nearly half of the patients were found to have dental hypoplasia, while less than one-tenth had hypogonadism. This discrepancy may be attributed to insufficient medical history documentation and evaluation, as patients often do not voluntarily disclose this information (Rydning et al., 2019). This highlights the significance of being attentive to these neurological symptoms when suspecting the disease clinically. Moreover, individuals with *POLR3A* gene-related syndrome may also suffer from visual and auditory impairments resulting from dysmyelination of the cranial nerves (Wolf et al., 2014).

*POLR3A* gene-related syndrome can also manifest with peripheral neuropathy phenotypes (Minnerop et al., 2017; Rydning et al., 2019), primarily affecting sensory nerve impairment, including affected deep sensation like vibration and proprioception, as well as superficial sensation. In this study, the patient did not experience symptoms such as numbness or weakness, and physical examination did not uncover any related abnormalities.

Based on literature reviewing (Table 1), this study found that the most common Variant sites of the *POLR3A* gene are c.1909 + 22G>A, c.2554A>G, and c.1771-6C>G. Patients with Variants at the c.1771-6C>G site have a younger average age of onset compared to those with c.1909 + 22G>A and c.2554A>G. The majority of patients with c.2554A>G are female. In terms of initial symptoms and clinical manifestations, patients with c.1909 + 22G>A primarily present with gait abnormalities, followed by ataxia, dysarthria, and nystagmus. Patients with c.2554A>G typically present with motor dysfunction and developmental abnormalities of teeth as initial symptoms, followed by worsening symptoms such as cognitive impairment, dental abnormalities, ataxia, and swallowing difficulties. Three patients also experiencing significant hypogonadism; patients with c.2554A>G mainly present with motor dysfunction and dysarthria as initial symptoms, followed by developmental abnormalities of teeth, ataxia, tremors, and abnormal muscle tone. The most frequent genotype among POLR3A-related mutations is the c.1909 + 22G>A variant in a compound heterozygous state (Ruggiero et al., 2020). This specific genotype is associated with a consistent and distinct clinical phenotype, characterized by late-onset spastic ataxia, hyperintensity of the SCP on MRI, and spinal cord atrophy. Commonly accompanying features include dental abnormalities, motor tremor, muscle wasting, dysarthria, pes cavus, ocular involvement, thinning of the corpus callosum, dystonia, and polyneuropathy. Notably, there is an absence of hypomyelinating leukodystrophy (Morales-Rosado et al., 2020).

Our review of the literature further confirms that patients carrying the c.1909 + 22G>A variant primarily exhibit SCP hyperintensity without evidence of hypomyelination, a finding that significantly distinguishes them from patients with other *POLR3A* variants. This strong genotype-phenotype correlation was substantiated by Di Donato et al., who demonstrated that the c.1909 + 22G>A variant, in combination with a second “variable” *POLR3A* mutation, is specifically associated with juvenile- or adult-onset spastic ataxia accompanied by SCP hyperintensity and spinal atrophy (Di Donato et al., 2022). Consistent with these observations, Minnerop et al. also reported that neuroimaging in patients with the c.1909 + 22G>A variant consistently reveals T2/FLAIR hyperintensity in the SCP, in the absence of significant hypomyelination. These collective findings underscore the unique clinico-radiological profile associated with this recurrent POLR3A variant (Minnerop et al., 2017).

The spectrum of diseases related to the *POLR3A* gene is wide and heterogeneous. Currently, there is no research clearly demonstrating the correlation between genotypes and phenotypes related to this gene (Minnerop et al., 2017; Rydning et al., 2019). However, haplotype analysis suggests that patients carrying the c.1378_1380del variant at the locus show more prominent neurological symptoms (Rydning et al., 2019).

In 2019, Siri L. Rydning and colleagues (Rydning et al., 2019) identified 10 cases of *POLR3A* gene biallelic Variants in 322 Norwegian patients with autosomal recessive or sporadic cerebellar ataxia or hereditary spastic paraplegia. Among these cases, 9 out of 10 had a c.1909 + 22G>A Variant in the intronic region, which results in the presence of a premature termination codon and further activates the disease-causing nonsense-mediated mRNA decay (NMD) mechanism.

The Variant site identified in the first individual studied is c.1771-6C>G. Azmanov et al. theorized that this variant may result in abnormal splicing, potentially causing exon 14 skipping and premature termination of the amino acid sequence.This could lead to a decrease in the full-length transcript level of *POLR3A* and potentially result in a deficiency of certain Pol III proteins (Azmanov et al., 2016). Additionally, abnormal Pol III metabolism could lead to a disruption in protein homeostasis and instability in the protein control system, which is similar to what is observed in neurodegenerative diseases (Azmanov et al., 2016).

The brain MRI images resulting from the c.1771-6C>G Variant differ from the previously reported white matter involvement, cerebellar and brainstem atrophy in patients with *POLR3A*-related HDL 7. They often present with specific involvement of the striatum and red nucleus (Azmanov et al., 2016). Compared with pedigrees with heterozygous Variants at the same site in the literature, the probands in this study had a slightly later onset age, and symptoms such as intellectual disability and tremors appeared later.The literature also indicates that the average age of onset in patients with homozygous Variants was significantly later than that in those with heterozygous Variants, and the radiological manifestations appeared milder in the former group. Takuya Hiraide and others (Hiraide et al., 2020) reported the brain MRI characteristics of three patients with compound heterozygous Variants of the *POLR3A* gene c.1771-6C>G from two families. They showed only brain atrophy and abnormal signals in the striatum, without white matter dysmyelination. In a report by Azmanov DN and others (Azmanov et al., 2016), three cases of homozygous Variant at the c.1771-6C>G site were documented, showing no diffuse white matter abnormalities on brain MRI. They also show that brain atrophy or signal abnormalities can be restricted to the striatum and may be subtle or absent on conventional MRI, especially in homozygous individuals who can present with a milder, later-onset radiological phenotype. Therefore, we speculate that the Variant at the c.1771-6C>G site may result in a relatively “benign” clinical phenotype.

In terms of cranial imaging, diseases related to the *POLR3A* gene may present with typical diffuse white matter abnormalities on brain MRI (Vrij-van et al., 2017). In addition, other manifestations such as abnormal signals in the cerebellar peduncles (Minnerop et al., 2017; Rydning et al., 2019), thinning of the corpus callosum, thinning of the cervical cord, and cerebellar atrophy may also be observed. According to the literature review, it was found that only one-third of patients exhibit typical diffuse white matter abnormalities on MRI. La P et al. proposed that diffuse white matter abnormalities are not a necessary imaging feature of *POLR3A* gene-related diseases, and introduced a new imaging pattern of selective involvement of the corticospinal tracts in 2016 (La et al., 2016). The pattern of selective involvement of the posterior limb of the internal capsule was also confirmed in patients reported by Siri L, Rydning, and others (Rydning et al., 2019).

Furthermore, the cranial MRI performed upon the patient’s admission revealed multiple brain infections and the presence of abscesses. It is currently unclear whether there is a direct correlation between the brain abscess and the Variant in the gene locus. Currently, there is no direct evidence establishing a causal relationship between *POLR3A* variants and brain abscess formation in this study. We hypothesize that *POLR3A* may contribute to brain abscess pathogenesis through the following mechanisms: First, *POLR3A* variants lead to dysfunctional RNA polymerase III (Pol III), reducing tRNA synthesis in immune cells and impairing the translation efficiency of antimicrobial proteins and cytokines (e.g., CXCL10). This is accompanied by suppressed expression of immune mediators such as IFN-β, thereby increasing cellular susceptibility to viral infection. Collectively, these alterations compromise both peripheral immune bactericidal capacity and the local immune microenvironment within the central nervous system, hindering effective pathogen clearance (Carter-Timofte et al., 2018; Ramanath et al., 2020). In support of this, Damian et al. demonstrated that inhibition of Pol III activity severely impairs macrophage function by delaying phagocytosis and suppressing cytokine secretion (Graczyk et al., 2015). Second, Pol III dysfunction may disrupt the synthesis of tight junction proteins (e.g., occludin, claudin-5) and mitochondrial metabolic proteins in vascular endothelial cells, thereby increasing blood-brain barrier permeability and reducing endothelial repair capacity, which facilitates bacterial invasion into brain tissue (Gao et al., 2023). Third, insufficient tRNA production may lead to the accumulation of cerebral amino acids (e.g., glutamate, aspartate), providing an energy source for bacterial proliferation and promoting the progression of infection into abscess formation (Li et al., 2024). Nevertheless, the possibility that brain abscess occurrence is not directly linked to *POLR3A* gene dysfunction cannot be excluded, warranting further investigation for validation.

In conclusion, this study presents a case of a *POLR3A*-related syndrome and integrates previous literature to advance the genetic understanding of this disorder. We propose that the *POLR3A* variants may be associated with a previously unreported phenotype characterized by cerebral abscess. Furthermore, cerebral atrophy was identified as the most consistent neuroimaging feature across all studied *POLR3A* variants. These findings highlight the importance of enhanced clinical vigilance for intracranial infections—such as fever or headache—in individuals carrying the *POLR3A* variant, and suggest that early MRI screening could facilitate timely antimicrobial treatment.

## Data Availability

The original contributions presented in the study are included in the article/Supplementary Material, further inquiries can be directed to the corresponding authors.
