# Supplementary material for: POLR3A-related syndrome complicated with cerebral abscesses: a case report and literature review
Source: Front Genet. 2026 Feb 5;17:1668022. doi: 10.3389/fgene.2026.1668022 (PMC12916066; doi:10.3389/fgene.2026.1668022)
Supplement: Supplementary file 1 [file Table1.doc]

**Tab.1 Literature review of probands with *POLR3A* gene-related syndrome probands previously reported.**

| Family serial number | Age at onset (y.) | Disease course (y.) | Gender | Symptom at onset | Clinical preservation and symptom | Brain MRI | Variant 1 | Variant 2 | Author, year |
| --- | --- | --- | --- | --- | --- | --- | --- | --- | --- |
| 1 | 12 | 43 | M | gait clumsiness, gait clumsiness  and recurrent falls | gait ATX, limb ATX, SA (gait), CD, head titubation, NYST, DENT | mild cerebellar atrophy | c.1909+22G>A | c.3337-11T>C | Avi Fellner, 2021^[29]^ |
| 2 | 16 | 2 | F | gait instability, coughing when drinking water | hypogonadism, CI, gait ATX | myelin sheath dysplasia in bilateral cerebral hemisphere, brain atrophy, thin corpus callosum, small pituitary, uneven enhancement, ventricular enlargement | c.3013C>T | c.1757C>T | Haojun Yang, 2023^[30]^ |
| 3 | 18 | 22 | M | gait clumsiness | gait ATX, SA (gait), lower limb ATX | significant abnormal signals in bilateral superior cerebellar peduncle and midbrain | c.1909+22G>A | c.1787C>T | Jon Infante, 2020^[31]^ |
| 4 | 30 | 18 | M | hand tremor | gait ATX, SA (gait), limb ATX, mild CD, extensor plantar | significant abnormal signals in bilateral superior cerebellar peduncle and midbrain | c.1909+22G>A | c.592G>T |
| 5 | 30 | 30 | F | gait clumsiness | gait ATX, SA (gait), lower limb ATX | significant abnormal signals in bilateral superior cerebellar peduncle and midbrain | c.1909+22G>A | c.1993dupT |
| 6 | 13 | 45 | F | gait linstability | gait ATX, SA (gait), limb ATX, mild CD, extensor plantar, DENT | significant abnormal signals in bilateral superior cerebellar peduncle and midbrain | c.1909+22G>A | c.646-687_1185+844del |
| 7 | 18 | 35 | M | gait clumsiness | gait ATX, SA (gait), limb ATX | significant abnormal signals in bilateral superior cerebellar peduncle and midbrain | c.1909+22G>A | c.646-687_1185+844del |
| 8 | 27 | 5 | F | gait clumsiness | gait ATX, SA (gait), lower limb ATX, extensor plantar | significant abnormal signals in bilateral superior cerebellar peduncle and midbrain | c.1909+22G>A | c.646-687_1185+844del |
| 9 | 0.8 | 1.7 | F | reduced motor ability, nystagmus, motor ataxia, dysarthria, spastic tetraplegia | ATX, NYST, CI, CD, SPAST-TET, DENT, polytrichia, hypermyotonia, DYSPHAG | cerebella, corpus callosum atrophy, white matter hypomyelination | c.1771-6C>G | c.2611del | Shuiyan Wu, 2019^[32]^ |
| 10 | 0.6 | 0.9 | M | developmental delay | DD, SPAST-PARA, MYODYS, progeroid facial appearance | NA | c.2005C>T | c.1771-7C>G | Purvi Majethia, 2021^[33]^ |
| 11 | 0.5 | 10.5 | F | developmental delay | DD, mobility limitation, ATX, aphasia, severe mental retardation, visual dysplasia, stereotypic hand movements, active tendon reflex, hypermyotonia, mild SA | cerebella, corpus callosum atrophy, white matter hypomyelination | c.346A>G | c.1745G>A | Mohamed Khalifa, 2015^[34]^ |
| 12 | 30 | 36 | F | progressive gait disorder | gait ATX, SPAST-PARA (gait), hypermyotonia (Babinski sign positive) | cerebellum and cervical spinal cord atrophy | c.1909+22G>A | c.3839dupT | Lucia Ruggiero, 2020^[35]^ |
| 13 | 2 | NA | M | NA | DD, NYST, CI, TR, upper motor neuron signs, cerebellar signs, DENT | NA | c.1674C>G | c.3742insACC | Genevie`ve Bernard, 2011^[36]^ |
| 14 | 1 | NA | M | NA | DD, seizure, optic atrophy, NYST, DYSPHAG, TR, upper motor neuron signs, cerebellar signs, hypersalivation | NA | c.2015G>A | |
| 15 | 3 | NA | M | NA | CI, optic atrophy, NYST, DYSPHAG, upper motor neuron signs, cerebellar signs, hypersalivation | NA | c.2015G>A | |
| 16 | 5 | NA | F | NA | CI, optic atrophy, upper motor neuron signs, cerebellar signs, hypersalivation | NA | c.2015G>A | |
| 17 | 13 | NA | F | NA | CI, seizure, DYSPHAG, upper motor neuron signs, cerebellar signs, hypersalivation | cerebellar atrophy, white matter hypomyelination | c.2554A>G | c.2711-1G>A |
| 18 | 0 | NA | F | NA | DD, CI, DYSPHAG, upper motor neuron signs, cerebellar signs, hypersalivation | cerebellar atrophy, white matter hypomyelination | c.2324A>T | c.1114G>A |
| 19 | 13 | NA | M | NA | CI, DENT, vertical gaze limitation, DYSPHAG, upper motor neuron signs, cerebellar signs | cerebellar atrophy, white matter hypomyelination | c.2830G>T | c.3013C>T |
| 20 | 12 | NA | F | NA | CI, DENT, vertical gaze limitation, DYSPHAG, upper motor neuron signs, cerebellar signs | cerebellar atrophy, white matter hypomyelination | c.2554A>G | c.2711-1G>A |
| 21 | 1 | NA | F | NA | CI, DD, DENT, upper motor neuron signs, cerebellar signs, TR | cerebellar atrophy, white matter hypomyelination | c.4006C>T | c.1907C>A |
| 22 | 12 | NA | F | NA | CI, upper motor neuron signs, cerebellar signs, TR | NA | c.2003+18G>A | |
| 23 | 3 | NA | F | NA | CI, DD, DENT, upper motor neuron signs, cerebellar signs, TR | NA | c.418C>T | c.2554A>G |
| 24 | 2 | NA | M | NA | CI, DD, DENT, vertical gaze limitation, upper motor neuron signs, cerebellar signs, TR, hypersalivation | NA | c.2171G>A | |
| 25 | 5 | 1 | F | developmental delay | local lipoatrophy, alopecia areata, osteopenia, progeroid facial appearance, DENT | NA | c.3568C>T | c.3337-11T>C | Sehime Gulsun Temel, 2020^[37]^ |
| 26 | 1 | 28 | F | esotropia and action tremor | ATX, CD, DENT, dysaudia, NYST, intention TR, postural seizures | white matter hypomyelination, brainstem, cerebellum and corpus callosum atrophy | c.930G>C | c.2411T>C | Keiko Shimojima, 2013^[38]^ |
| 27 | 19 | 15 | F | gait disorder, amenorrhea, progressive cognitive impairment | ATX, CD, NYST, limb SA, DYSK | the corpus callosum, cortex / subcortex, brain stem and cerebellum atrophy, supratentorial ventricular system dilatation, white matter hypomyelination. | c.2325C>G | c.2554A>G | Rosa Campopiano, 2020^[39]^ |
| 28 | 15 | 26 | F | dental developmental abnormalities, amenorrhea | amenorrhea, movement disorder, DENT, gait ATX, cerebellar ATX, limb SA, tonic seizure, DYSPHAG, osteoporosis | white matter hypomyelination, brainstem, cerebellum and corpus callosum atrophy | c.2554A>G | c.2668G>T | Soma Furukawa, 2021^[40]^ |
| 29 | 8 | 0 | M | cerebellar dysarthria | CD, ATX, hypomyotonia, TR | bilateral symmetric atrophy, increased sig-  nal of the caudate nucleus and the putamen | C.1771-6C>G | c.791C>T | Takuya Hiraide, 2020^[41]^ |
| 30 | 1.5 | NA | F | dysstasia | gait ATX, CD, ophthalmoparesis, MYODYS, DENT, mandibular underdevelopment, hypomyotonia | diffuse brain atrophy | c.1771-6C>G | c.2671C>T |
| 31 | 18 | 35 | F | gait ataxia | gait ATX, ATX, CD, DD, DENT | white matter hypomyelination, corpus callosum, cerebellum atrophy, thoracic spinal cord thinning | c.928T>A | c.3295C>T | Ling Li, 2022^[11]^ |
| 32 | 1 | 2 | F | developmental delay | DD, coughing when drinking, mobility limitation, MYODYS | abnormal signals in lentiform nucleus, putamen and caudate nucleus | c.1980 G>C | c.1771‑6 C>G | Fang He, 2021^[9]^ |
| 33 | 1.3 | 2.7 | F | developmental delay | DD, seizure, MYODYS, NYST | abnormal signals in caudate nucleus, lentiform nucleus and bilateral paraventricular | c.2044C>T | c.1771‑7C>G |
| 34 | 0.5 | 1.5 | M | gait linstability | DD, gait ATX, DENT, CD, NYST, TR | diffuse abnormal signals in bilateral large and small cerebral hemisphere white matter area | c.3858C>A | c.3226G>A | Conglei Song, 2021^[42]^ |
| 35 | 5.7 | 0.3 | M | gait linstability, delayed development of language | DD, gait ATX, DENT, optic atrophy, NYST, intention TR | widely symmetrical white matter lesions on both sides of the cerebral hemisphere | c.1781T＞G | c.2693delT | Chao Liang, 2017^[43]^ |
| 36 | 0.3 | 36.7 | F | facial deformity | facial deformity, DD, hearing abnormality, DENT, severely cachexic appearanc | abnormal signal in cerebellar | c.3336G>A | | Davor Lessel, 2021^[44]^ |
| 37 | 1.5 | 15.5 | M | delayed psychomotor development and absence of language, gait ataxia | ATX, CD, DENT, DYSPHAG, NYST, gait ATX, ptosis | corpus callosum, cerebellum atrophy, white matter hypomyelination | c.1795C>A | c.328A>G | Antonino Musumeci, 2022^[45]^ |
| 38 | 1.5 | 4.5 | M | gait ataxia | dysontogenetic, seizure, MYODYS, DYSPHAG, CD | abnormal signal in striatum | c.1771-6C>G | c.4037G>A | Ali Nikkhah, 2022^[46]^ |
| 39 | 19 | 56 | M | gait ataxia | DYSK, gait ATX, NYST, CD, pyramidal signs, cerebellar signs, head TR, sensory peripheral neuropathy | NA | c.1909+22G>A | | Trevor M. Sytsma, 2021^[47]^ |
| 40 | 15 | 27 | F | developmental delay | ATX, DENT, amenorrhea | diffuse cortical atrophy, white matter hypomyelination | c.1911+18C>T | | Yi-Ming Yang, 2019^[48]^ |
| 41 | 0.5 | 1 | F | nystagmus | ATX, DD, DENT, NYST, hearing abnormality, mandibular underdevelopment | white matter hypomyelination, diffuse atrophy | c.2423G>A | | Vishal V. Tewari, 2018^[49]^ |
| 42 | 26 | 8 | F | gait ataxia | ATX, CD, mobility limitation, DENT, NYST, static TR | abnormal signals around the ventricle, frontal lobe and temporal lobe | c.4044C>G | c.1186-2A>G | Lei Sun, 2023^[50]^ |
| 43 | 4 | 29 | M | dyskinesia | hypogonadism, CI, DENT, cerebellar ATX, intelligence decline | cerebella, corpus callosum atrophy, white matter hypomyelination, cerebellar abnormal signal | c.2554A>G | c.3745A>C | Terao , 2012^[51]^ |
| 44 | 8 | 48 | M | cerebellar dysarthria, gait ataxia | ATX, movement disorder, NYST, bilateral symmetric rigidity (right hand/lower limb), right wrist gear phenomenon | a mild small brain, with bilateral symmetric atrophy of the caudate nucleus and putamen and associated increased signal, focal symmetrical signal changes in the medial red nucleus area and the third brain nerve axis, the white matter was of normal volume and signal | c.1771-6C>G | | AZ, 2016^[17]^ |
| 45 | 7 | 24 | M | speech disturbances | MYODYS, gait instability, DYSPHAG, dysmelia, intelligence decline, extensor plantar | bilateral symmetric atrophy and increased signal of the caudate nucleus and putamen, with prominence of the lateral ventricular frontal horns as a consequence, and focal bilateral symmetric signal change in the region of the medial red nucleus intra-axial course of the third cranial nerve | c.1771-6C>G | |
| 46 | NA | NA | NA | gait ataxia, cerebellar dysarthria, tremor | cerebellar TR, DENT | a selective involvement of the corticospinal tracts, which was particularly evident at the level of the posterior limbs ofthe internal capsule as T2-hyperintense signal | c.1048+1G＞A | c.128913A＞C | Roberta, 2016^[23]^ |
| 47 | NA | NA | NA | spasticity and  diplegic gait | cerebellar TR, pyramidal signs, SA, severe dystonic TR, DENT | a selective involvement of the corticospinal tracts, which was particularly evident at the level of the posterior limbs of the internal capsule as T2-hyperintense signal, focal, partially confluent, T2-hyperintense white matter abnormalities located in the deep frontal and parietal white matter, suggesting partial hypomyelination | c.2710 G＞A | |
| 48 | NA | NA | NA | spasticity and  diplegic gait | cerebellar TR, pyramidal signs, SA, severe dystonic TR, DENT | moderate to severe cerebellar atrophy was variably associated with nonspecific T2-hyperintense white matter abnormalities or thinning of the corpus callosum. Focal, partially confluent, T2-hyperintense white matter abnormalities located in the deep frontal and parietal white matter, suggesting partial hypomyelination | c.1771-6C＞G | c.3205C＞T |
| 49 | NA | NA | NA | NA | pyramidal signs, SA | moderate to severe cerebellar atrophy was variably associated with nonspecific T2-hyperintense white matter abnormalities or thinning of the corpus callosum.focal, partially confluent, T2-hyperintense white matter abnormalities located in the deep frontal and parietal white matter, suggesting partial hypomyelination | c.2381A＞C | c.-35C＞G |
| 50 | NA | NA | NA | gait ataxia, cerebellar dysarthria, tremor | pyramidal signs, SA, DENT | a selective involvement of the corticospinal tracts, which was particularly evident at the level of the posterior limbs of the internal capsule as T2-hyperintense signal | c.1909+22G＞A | c.2549A＞G |
| 51 | 14 | 51 | F | gait linstability | TRE-ATX, limbs tendon reflex weakened, lower limb weakness/atrophy, CD, limb ATX, foot deformity, postural TR, head/neck titubation, hypoesthesia, urinary urgency | SCP high signal, cervical spinal cord thinning, cerebellar hemisphere and vermis atrophy | c.1909+22G＞A | c.3655G>T | Siri L. Rydning, 2019^[7]^ |
| 52 | 12 | 35 | M | gait linstability | TRE-ATX, lower limb SA, limb tendon reflex reduction, lower limb weakness/atrophy, NYST, CD, limb ATX, CI, myopia, postural TR, head/neck titubation, hypoesthesia, urinary urgency, foot deformity | SCP high signal, cervical spinal cord thinning, cerebellar vermis atrophy | c.1909+22G>A | c.3655G>T |
| 53 | 17 | 27 | M | stiff legs | TRE-ATX, lower limb SA, limb tendon reflex reduction, lower limb weakness/atrophy, MYODYS, NYST, CD, limb ATX, postural TR, head/neck titubation | SCP high signal, cervical spinal cord thinning, cerebellar hemisphere and vermis atrophy | c.1909+22 G>A | c.3655G>T |
| 54 | 13 | 33 | M | gait linstability | cHSP, lower limb SA, limb tendon reflex reduction, lower limb weakness/atrophy, MYODYS, postural TR, limb ATX, head/neck titubation, hypoesthesia, CI, foot deformity | SCP high signal, cervical spinal cord thinning, cerebellar vermis atrophy | c.1909+22 G>A | c.3655G>T |
| 55 | 30 | 15 | M | gait linstability | TRE-ATX, lower limb SA, limb tendon reflex reduction, lower limb weakness/atrophy, NYST, upper limb ATX, DENT, hypoesthesia, urinary urgency, foot deformity | SCP high signal, cervical spinal cord thinning, cerebellar vermis atrophy | c.1909+22 G>A | c.3655G>T |
| 56 | 10 | 55 | F | clumsy | TRE-ATX, lower limb SA, limb tendon reflex reduction, lower limb weakness/atrophy, NYST, CD, limb ATX, MYODYS, head/neck titubation, DENT, myopia, postural TR, hypoesthesia | NA | c.1909+22 G>A | c.3655G>T |
| 57 | 11 | 46 | M | stiff legs | cHSP, lower limb SA, limb tendon reflex reduction, lower limb weakness/atrophy, NYST, lower limb ATX, DENT, myopia, hypoesthesia, urinary urgency, scoliosis | SCP high signal, cervical spinal cord thinning, cerebellar hemisphere and vermis atrophy | c.1909+22 G>A | c.1682G>A |
| 58 | 17 | 28 | M | gait linstability | TRE-ATX, lower limb SA, limb tendon reflex reduction, lower limb weakness/atrophy, NYST, CD, limb ATX, DENT, postural TR, MYODYS, myopia, hypoesthesia, urinary urgency, scoliosis, hypogonadism | SCP high signal, cervical spinal cord thinning, cerebellar vermis atrophy | c.1909+22 G>A | c.1378_ 1380del |
| 59 | 5 | 24 | F | gait linstability | TRE-ATX, limb tendon reflex reduction, NYST, limb ATX, DENT, MYODYS, hypoesthesia, foot deformity, head/neck titubation, CI | SCP high signal, cervical spinal cord thinning, cerebellar vermis atrophy | c.1909+22 G>A | c.1378_ 1380del |
| 60 | 4 | 41 | M | gait linstability | cHSP, lower limb SA, limb tendon reflex reduction, lower limb weakness/atrophy, CD, NYST, limb ATX, DENT, MYODYS, myopia, hypoesthesia, postural TR, head/neck titubation | SCP high signal, cervical spinal cord thinning, cerebellar vermis atrophy | c.1771-6 C>G | |

**Abbreviation:**

ATX: Ataxia; SA: Spasticity; TR: Tremor; CD: Cerebellar dysarthria; CI: Cognitive impairment; DENT: Dental abnormalities; DD: Developmental delay; NYST: Nystagmus; DYSPHAG: Dysphagia; SPAST-TET: Spastic tetraplegia; SPAST-PARA: Spastic paraplegia; MYODYS: Myodystonia; DYSK: Dyskinesia; CEREBELL-ATRO: Cerebellar atrophy; CORP-CALL-ATRO: Corpus callosum atrophy; CORP-CALL: Corpus callosum; WM-HYPOMYO: White matter hypomyelination; SCP: Superior cerebellar peduncles; TRE-ATX: Tremor-ataxia; cHSP: Complex hereditary ataxia and spastic paraparesis; F: Female; M: Male; NA: Not available
